# Incidence and prognosis of contralateral vocal fold paralysis after hemithyroidectomy in previously unoperated patients

**DOI:** 10.1093/bjsopen/zrad126

**Published:** 2023-11-13

**Authors:** Stefanie Sinz, Franziska Grafen, Walter Kolb, Jochen Rosenfeld, Thomas Clerici

**Affiliations:** Department of Surgery, Kantonsspital St.Gallen, St. Gallen, Switzerland; Private University of the Principality of Liechtenstein, Liechtenstein; Department of Surgery, Spital Limmattal, Schlieren, Switzerland; Department of Surgery, Kantonsspital St.Gallen, St. Gallen, Switzerland; Department of Otorhinolaryngology, Head and Neck Surgery, St. Gallen, Switzerland; Department of Surgery, Kantonsspital St.Gallen, St. Gallen, Switzerland

## Introduction

Hemithyroidectomy, the removal of either the right or left lobe of the thyroid gland, is one of the most common endocrine surgeries performed worldwide. The most frequently associated complications include damage to the recurrent laryngeal nerve (RLN) with consecutive vocal fold paralysis (VFP), damage to the external branch of the superior laryngeal nerve, bleeding, and parathyroid insufficiency (hypoparathyroidism)^1^. During initial unilateral hemithyroidectomy the contralateral thyroid lobe is neither explored nor dissected. Complications such as VFP of the contralateral is not expected because there is no possibility of direct, surgically induced harm to the RLN. Therefore, VFP of the contralateral, non-operated side after hemithyroidectomy is extremely rare and unexpected. Beyond some published case reports, hardly any data are available on the incidence and prognosis of contralateral VFP. To clarify the incidence rate and prognosis of contralateral VFP in hemithyroidectomy, the authors performed a single-centre retrospective cohort study based on prospectively collected data.

## Methods

The study population comprised all patients undergoing initial thyroid resection in a previously not preoperated neck between January 2012 and December 2021 at Cantonal Hospital St. Gallen, Switzerland. The operations were either performed or assisted by two specialized European union of medical specialists-certified endocrine surgeons with an individual annual caseload of approximatively 150 thyroid resections.

Patients underwent preoperative and postoperative video laryngoscopy by an independent ear nose throat (ENT) phoniatrician. While performing hemithyroidectomy, the contralateral side was never explored nor its RLN dissected. Intermittent intraoperative neuromonitoring (IONM) was used as standard of care. VFP was defined as any newly discovered hypomobile vocal fold at postoperative laryngoscopy^2^ and permanent VFP as persistent functional impairment 6 months after hemithyroidectomy. In patients with postoperative paralysis, follow-up laryngoscopy was usually performed after 4–6 weeks and 6 months to establish the recovery rate.

Baseline demographic data and other parameters potentially associated with recurrent nerve paralysis were retrieved from the hospital’s medical system.

## Results

During the study period, 1598 hemithyroidectomies were performed. A total of 235 patients who had secondary thyroid resections were excluded, leaving 1363 patients in the study for analysis. Patient characteristics are depicted in *Table 1*.

**Table 1:** Patient characteristics

**Sex**	
Male	326 (23.9%)
Female	1037 (76.1%)
**Age (years), median (range)**	55.3 (10.9–91.6)
**Indication for surgery**	
Compressive symptoms/goiter	52.9%
Excluding malignancy	34.9%
Hyperthyroidism	7.0%
Carcinoma	3.6%
Other	1.6%
**Surgical site**	
Right sided	53.6%
Left sided	46.4%
**Mallampati score (*n* = 20)**	
Mallampati I	17 (13 ipsilateral paralysis, 4 contralateral paralysis)
Mallampati II	3 (3 ipsilateral)
Mallampati III	0
Mallampati IV	0
**Performed laryngoscopy**	
Preoperative − Pre-existing recurrent nerve paralysis	1356 (99.5%) 3 (0.2%)
Postoperative − Postoperative recurrent nerve paralysis	1350 (99.1%) 42 (3.1%)
**Other complications**	
Postoperative haemorrhage	7 (0.5%)
Wound revision	7 (0.5%)
Other complications not associated with surgery (for example, cardiac, pulmonary, positioning injury)	6 (0.5%)
**Intraoperative neuromonitoring in overall patients with postoperative recurrent nerve dysfunction (*n* = 42)**	
Complete loss-of-signal	16 (38.1%)
Lower amplitude at the end of surgery	4 (9.5%)
No adequate signal measurable	8 (19.1%)
Normal signal	14 (33.3%)
**Intraoperative neuromonitoring in patients with contralateral recurrent nerve dysfunction (*n* = 8)**	
Complete loss-of-signal on operated site	0 (0%)
Lower amplitude at the end of surgery	1 (12.5%)
No adequate signal measurable	0 (0%)
Normal signal on operated site	7 (87.5%)

**Fig. 1:**
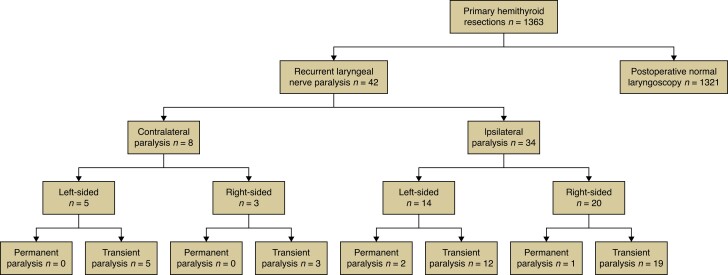
Patient distribution

At postoperative laryngoscopy eight patients (0.6 per cent) presented with contralateral recurrent paralysis; all were female. Their median age was 64.0 years (range: 28.6–79.1 years). Five cases of VFP were localized on the left side and three on the right side. Six patients had been operated on for goiter size or compressive symptoms, whereas two patients had been operated on for hyperthyroidism. The affected cases were evenly distributed between both responsible surgeons. Affected patients were symptomatic to a varying degree, from severe hoarseness to barely audible voice changes. All patients received speech therapy and periodic laryngoscopy to evaluate the course of vocal fold palsy. Clinical symptomatology and VFP at laryngoscopy completely resolved in all eight cases within 6 months. *Figure 1* depicts patient distribution.

## Discussion

As a normal voice does not exclude postoperative VFP, and laryngoscopy indicated selectively for voice changes underestimates the true VFP rate^3^, endocrine surgical societies^4–9^ and outcome registries (EUROCRINE^®^)^10^ generally consider preoperative and postoperative laryngoscopy mandatory for outcome assessment after thyroidectomy.

In terms of process quality, a control laryngoscopy before discharge allows for almost complete detection of symptomatic as well as asymptomatic patients with VFP. With a postoperative laryngoscopy rate of 99.5 per cent, the follow-up of this study cohort was almost complete. As the examination was performed by an ear, nose and throat (ENT) phoniatrician, hardly any ipsilateral or contralateral VFP would have remained undetected in the present study. This clearly adds to the credibility and correctness of the noted incidence of 0.6 per cent contralateral VFP on the non-operated side. The authors assume that the detection rate for contralateral VFP would be significantly lower or this type of complication hardly ever detected by less-qualified examiners or a lower postoperative laryngoscopy rate. Therefore, it is not surprising that contralateral VFP on the non-operated side is hardly mentioned or specifically investigated in the published literature.

If postoperative laryngoscopy for the assessment of vocal fold mobility were to be completely replaced by the measurement endpoints of the Intermittent intraoperative neuromonitoring (IONM), contralateral VFP would not be detected because, by definition, it would not be possible to perform the IONM if dissection of the nerve of the contralateral side is omitted.

Knowing that the VFP in all eight patients recovered completely, both clinically and laryngoscopically, one might question the importance of postoperative laryngoscopy in these patients in the absence of any long-term consequences. However, symptomatic patients with endoscopically confirmed paralysis certainly benefit from logopaedic care even in cases of temporary paralysis. From this, it may be deduced that, if the outcome of recurrent nerve function is controlled exclusively by IONM endpoints, at least every patient with postoperative dysphonia must be referred to laryngoscopy by an ENT specialist. Basically, the same practice must be demanded for patients who must undergo early postoperative completion thyroidectomy when diagnosed with well-differentiated thyroid carcinoma. The laryngoscopically confirmed diagnosis of VFP and clinical need for logopaedic follow-up would certainly legitimize the delay of completion thyroidectomy with good reason.

As all patients with contralateral VFP were female, gender may be a risk factor, possibly due to the smaller larynx in females, as it is also causative in intubation granuloma^11^. The pathomechanism behind this pathology is evoked due to the minor dimensions of the larynx configuration in females, with a larger contact area for the intubation tube with the airway mucosa^12,13^. Oversized tubes may cause pressure damage to the surrounding structures with subsequent paralysis of the recurrent nerves^14^. Therefore, a smaller tube size should be considered in small females.

Undoubtedly, individual surgical experience is a crucial factor related to the probability of complications. Based on the high annual case numbers of both surgeons involved in the current study, it is not surprising that no difference was found in this regard. The affected cases were evenly distributed between both responsible surgeons.

Of the most commonly mentioned pathogenetic mechanisms for recurrent nerve damage^15,16^, heat, contusion of the nerve, mechanical neurolysis, or compromised blood supply in the absence of surgical exposure can be definitively ruled out. Although it is difficult to imagine a plausible explanation, indirect damage by means of traction on the RLN cannot be excluded with absolute certainty.

Because the cause of VFP on the non-operated side of the neck cannot be attributed to surgical trauma, it is reasonable to evaluate whether the damage to the recurrent nerve may occur during the course of manipulations during intubation (hyperextension, intubation). This hypothesis is supported by VFP also being described, although rarely, after general anaesthesia that was not performed for an operation in the neck^17–19^. However, if intubation was a causative factor for non-surgical VFP, it would be reasonable to hypothesize that a technically difficult intubation would be more likely to result in non-surgical VFP. Other manipulative factors, such as the degree of reclination during intubation or initial positioning of the tube’s cuff, may also represent contributing factors to a non-surgical VFP.

In summary, this study suggests that female gender might be a risk factor for a contralateral VFP, but the potential pathogenic mechanisms of this complication remain elusive. Importantly, all contralateral VFP cases recovered completely both clinically and videoendoscopically, which is an important piece of information for the affected patient and their surgeons when this rare complication occurs.

## Data Availability

The data that support the findings of this study are available on request from the corresponding author (S.S.).
